# Supplementary material for: Pre-hospital interventions in snakebite: A telephonic survey and follow up investigating snakebite envenoming from a tertiary care centre in Coastal Karnataka
Source: PLoS Negl Trop Dis. 2025 Dec 19;19(12):e0013334. doi: 10.1371/journal.pntd.0013334 (PMC12742758; doi:10.1371/journal.pntd.0013334)
Supplement: S1 Text — Annexure 1: IEC approval of the study. Annexure 2: Telephonic Survey Tool. Annexure 3: Health Education Content. Annexure 4: Demographics of Non-responders. Annexure 5: Definitions of Healthcare facility and Disabilities. (DOCX) [file pntd.0013334.s001.docx]

**SUPPORTING DOCUMENT**

**APPENDIX 1**

**INSTITUTIONAL ETHICS COMMITTEE APPROVAL**


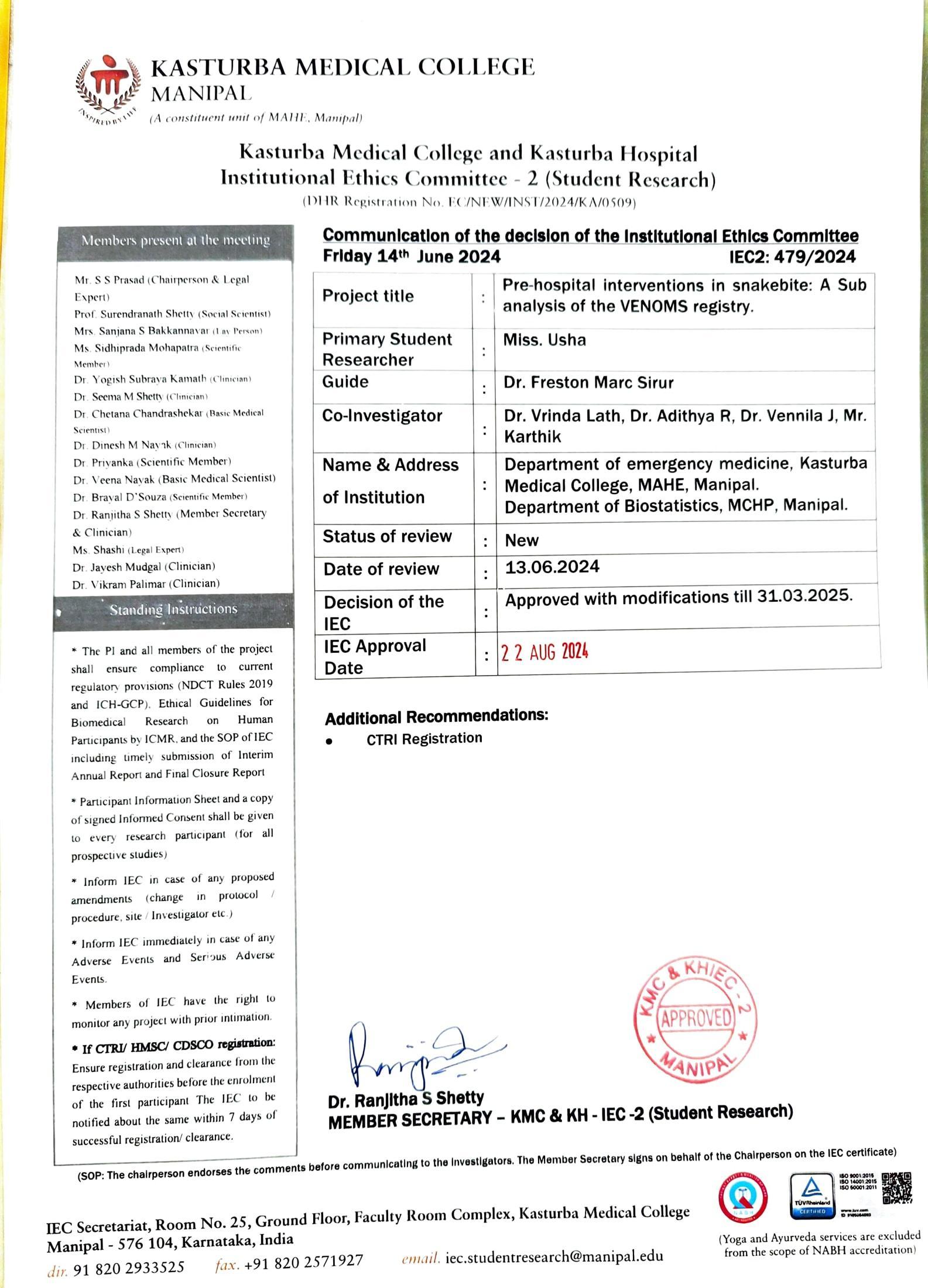


**APPENDIX 2**

Telephonic survey Tool

- Call answered by: Patient/ Bystander / Family / Friend
- Were you present when the incident happened? YES/ NO

(If NO, then the interviewee will be asked to direct to the person who was aware or present during the incident. Their contact will be collected, verbal consent will be obtained, and the questionnaire will be delivered. An attempt will be made to contact them in-person, if within the same district.

1. Who was the first responder following the snakebite incident?

- General public
- Family members
- Colleagues
- Healthcare workers
- Asha workers
- Forest officers
- Fire department
- Police department
- No first responder

Others: __________________

1. Was any first aid treatment given before reaching healthcare facility?

- YES
- NO

| Non-recommended first aid response | Recommended first aid response |
| --- | --- |
| - Tourniquet - Incision - Herbal decoction - Ayurvedic therapy - Topical herbal application - Burning - Bloodletting - Cauterization   If others, specify: _____________ | - Identify the emergency - scene safety - Reassurance - Immobilization - Recovery position - Supplemental oxygen - Rescue breaths - Cardiopulmonary resuscitation - Capturing the evidence - Immediate transfer to healthcare facility   If others specify: _______________ |

1. Following the snakebite, how many healthcare facilities were visited before arriving at KH hospital? (Specify the level)?

| 1^ST^ LEVEL   - PHC - CHC - District hospital - Private hospital - Private clinic - Private referral Centre - Government medical college & hospital | 2^ND^ LEVEL   - PHC - CHC - District hospital - Private hospital - Private clinic - Private referral Centre - Government medical college & hospital | 3^RD^ LEVEL   - PHC - CHC - District hospital - Private hospital - Private clinic - Private referral Centre - Government medical college & hospital | 4^TH^ LEVEL   - PHC - CHC - District hospital - Private hospital - Private clinic - Private referral Centre - Government medical college & hospital |
| --- | --- | --- | --- |

1. After the bite, how long did it take to reach the first healthcare facility? __________

Type of Road:

- Mud road (MOTORABLE)
- Footpath /dirt trail
- Tar road
- No accessible road

1. What was the mode of travel to receive first aid? SINGLE / MULTIPLE

| 1^ST^ LEVEL   - Walk - Run - Bullock cart - Bus - Bike - Autorickshaw - Car - Bicycle - Truck - Ambulance - Others: __________ | 2^ND^ LEVEL   - Walk - Run - Bullock cart - Bus - Bike - Autorickshaw - Car - Bicycle - Truck - Ambulance - Others: __________ | 3^RD^ LEVEL   - Walk - Run - Bullock cart - Bus - Bike - Autorickshaw - Car - Bicycle - Truck - Ambulance - Others: __________ | 4^TH^ LEVEL   - Walk - Run - Bullock cart - Bus - Bike - Autorickshaw - Car - Bicycle - Truck - Ambulance - Others: __________ |
| --- | --- | --- | --- |

1. Was a healthcare worker present in the ambulance accompanying the patient?

YES / NO / NOT APPLICABLE

If YES,

- Emergency medical technician
- Nurse
- Asha worker
- First aid trained driver
- Doctor
- Housekeeping staff
- Forest officer
- Police department
- Fire department
- Others: ______________________

1. What medical interventions were done in the ambulance?

(Checkboxes to serve as guide for interviewer)

| - None - IV cannulation - LMA / I-Gel - Bladder catheterization - Endotracheal intubation - Nasogastric tube - OTHERS: ___________ | - IV fluids - Supplemental oxygen - Immobilization - IV drugs - Cardiopulmonary resuscitation |
| --- | --- |

1. After the snakebite incident, what happened to the snake that bit?

- Kill the snake
- Burn the snake
- Live snake taken to hospital
- Called forest officer
- Snake went away
- Snake not seen
- Brought the dead specimen to hospital
- Others: ________________________

1. Does the patient have any disability currently?

- Non – healing ulcer
- Amputation
- Gangrene
- Renal failure—HD / no HD
- CVA
- None
- Others: _________________________

1. What types of ambulance services are available in your area?

- Private ambulance – BLS / ALS
- Government ambulance – BLS / ALS
- Bike ambulance
- Not available

1. What were the challenges that were encountered in accessing medical care or reaching the hospital following the snakebite incident?

- Lack of ambulance service
- Accessibility
- Trained provider
- Lack of awareness
- No telephone
- Non-availability of ASV
- No motorable road
- No healthcare worker at PHC
- No snakebite treating expert in local healthcare facility

1. Name & location of the nearest healthcare facility. _______________________
2. I would like to ask certain steps on first aid can you confirm if its correct or wrong.
3. Application of a tourniquet - YES / NO
4. Offering victim reassurance - YES / NO
5. Immobilization of the affected limb - YES / NO
6. Consideration of traditional or Ayurvedic healing methods - YES / NO
7. Consideration of incision and bloodletting - YES / NO
8. Anti-snake venom - YES / NO
9. Capturing evidence - YES / NO
10. Killing & burning the snake – YES / NO
11. Calling for help (eg: Asha worker) – YES / NO

**APPENDIX 3**

**HEALTH EDUCATION CONTENT**

Brief health education given to the patients post data collection

- In case of a snakebite, take responsibility for the appropriate response:

1. Recognize the emergency.
2. Move the patient away from the danger, assess their condition.
3. Calm the patient and offer reassurance to help manage their stress and anxiety.
4. Determine the severity of the situation and decide the quickest and most appropriate mode of. transport to the hospital.
5. Contact emergency services immediately, inform them of the situation, and identify the nearest hospital equipped to handle snakebites.
6. Document the incident by taking photos or videos of the snake if safe to do so or mentally note details of the event to assist the treatment.
7. Ensure the patient is transferred to an appropriate medical facility and communicate essential information to the treating physician.

- Avoid inappropriate handling of snakes. Avoid inappropriate bite site management like making incisions, scraping the bite site, or using tourniquets, as these actions can do more harm than good. Do not attempt to kill the snake.

To prevent snakebites, use a torch when walking at night, use protective footwear such as boots, avoid open toilets, and do not sleep outside without mosquito nets. Keep your house and surroundings clean, free of rodents. Use well demarcated paths and check before walking

**VALIDATION PROOF**

The health education session was validated by experts independent of the study team.
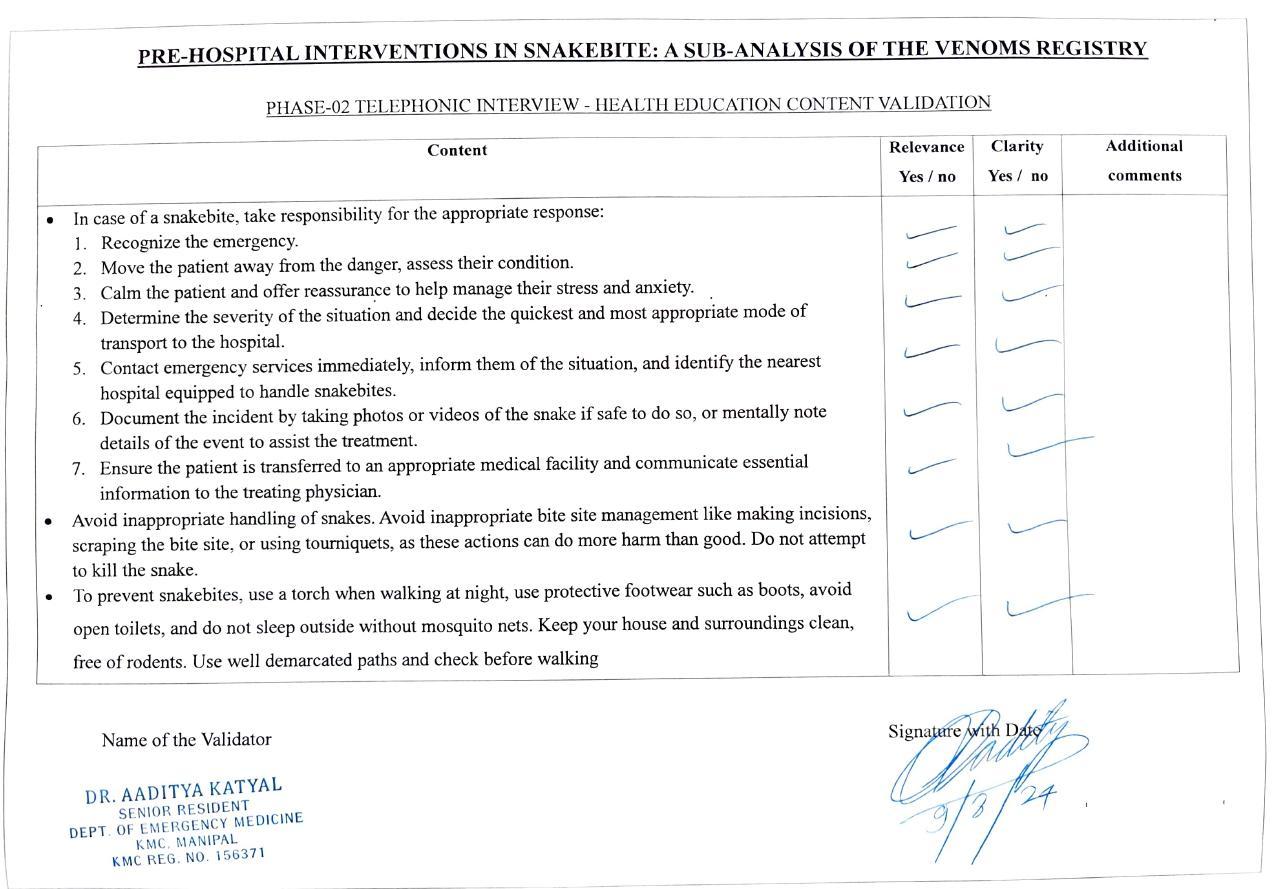


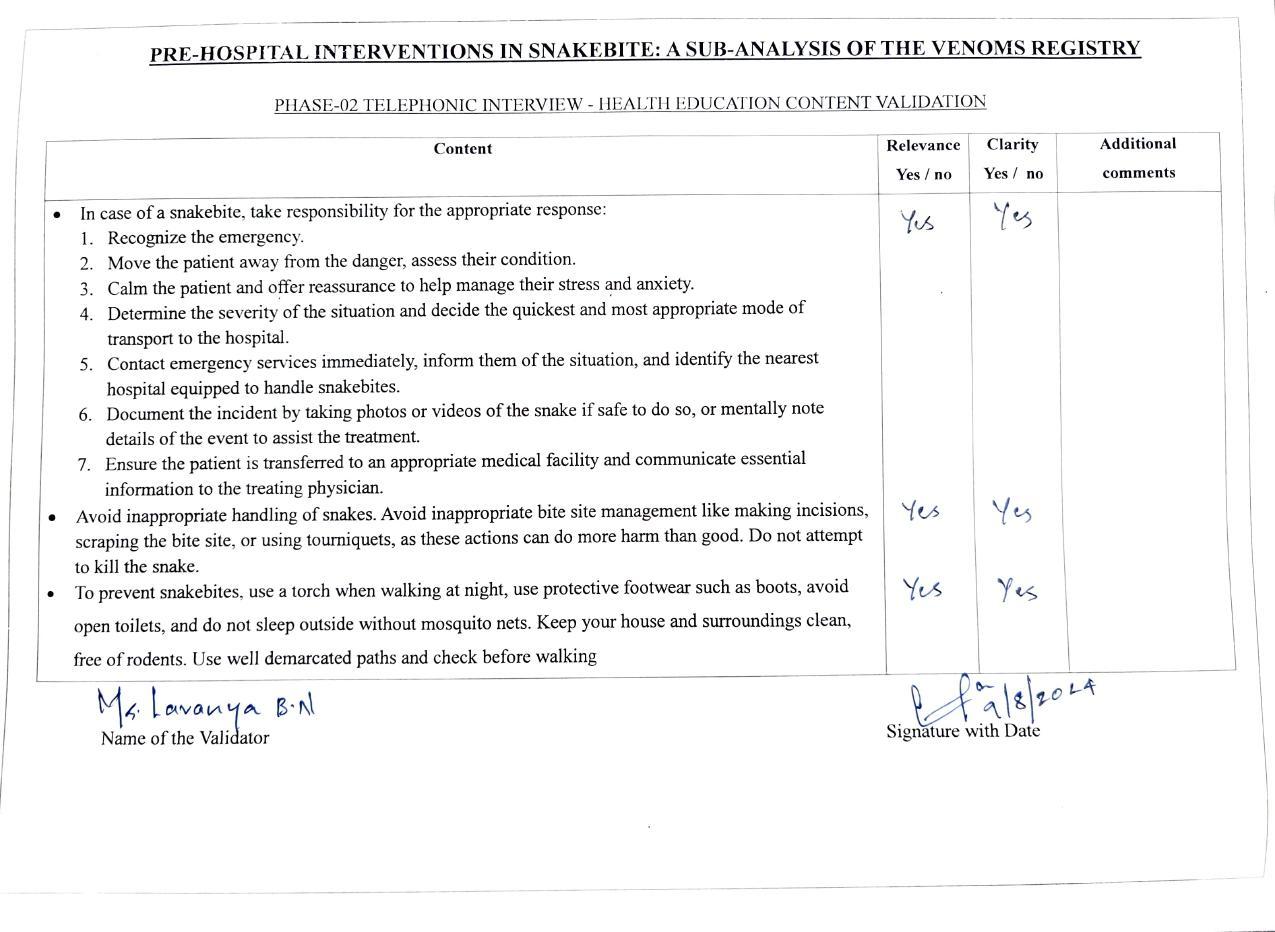


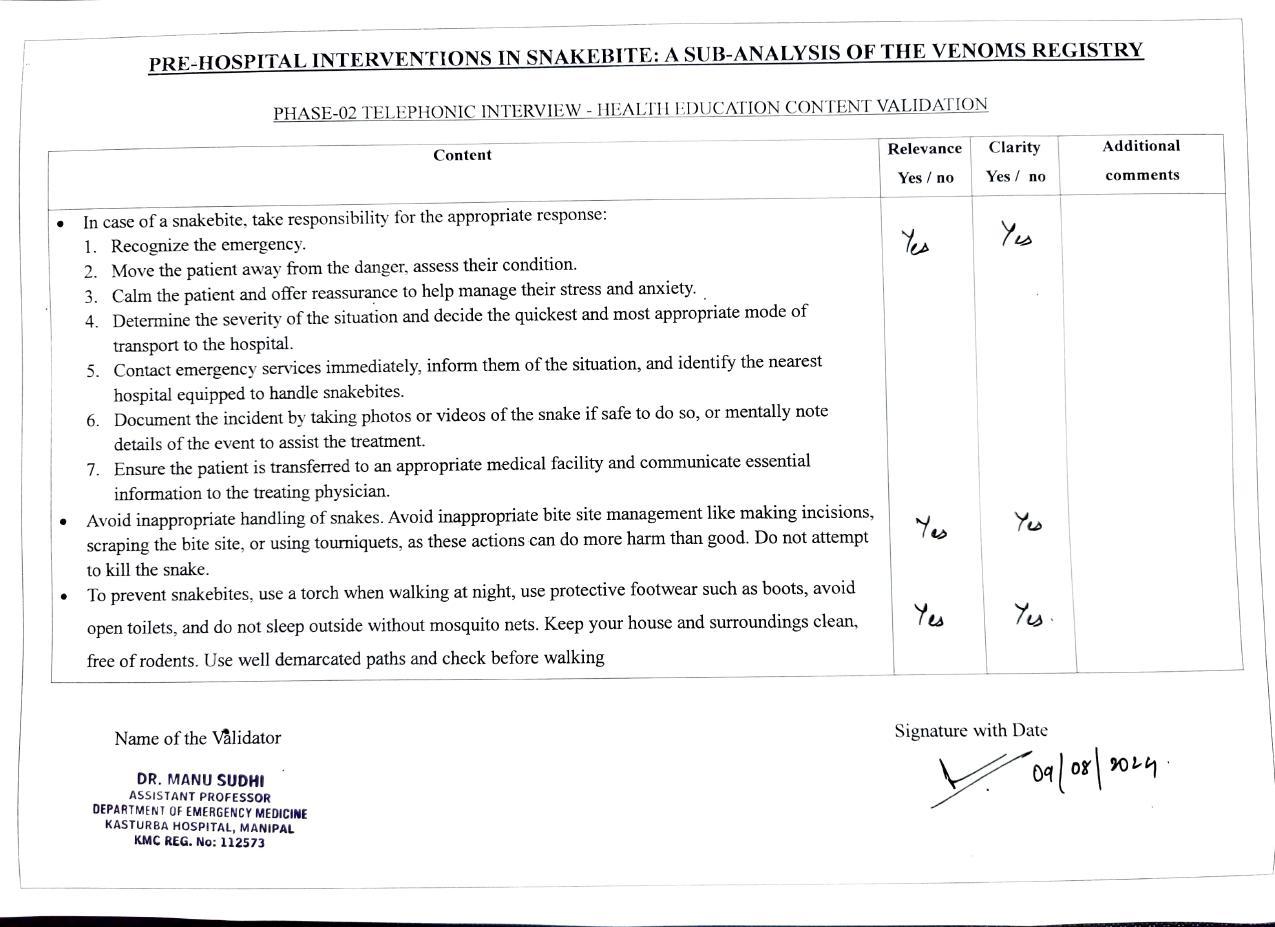


**APPENDIX 4**

**Basic demographics and bite details of the non-responders**

| Gender | | Method of identification | |
| --- | --- | --- | --- |
| Male | 202 (66.2%) | Photographic evidence | 35 (11.5%) |
| Female | 103 (33.8%) | Dead/live specimen brought to hospital | 28 (9.2%) |
| Age | | Photographic identification | 69 (22.6%) |
| 0-10 | 11 (3.6%) | Syndromic Identification | 173 (51.6%) |
| 11-20 | 21 (6.9%) | **Species identified** | |
| 21-30 | 63 (20.7%) | Russell’s viper | 58 (19.0%) |
| 31-40 | 46 (15.1%) | Spectacled cobra | 35 (11.5%) |
| 41-50 | 64 (21.0%) | Saw scaled viper | 3 (1.0%) |
| 51-60 | 64 (21.0%) | Common Krait | 8 (2.6%) |
| 61-70 | 31 (10.2%) | Malabar pit viper | 6 (2.0%) |
| 71-80 | 4 (1.3%) | Hump nosed pit viper | 32 (10.5%) |
| 81-90 | 1 (0.3%) | Non-venomous | 74 (23.3%) |
| Season of bite | | Syndromic-hemotoxic | 67 (22%) |
| Summer | 74 (24.3%) | Syndromic- Neurotoxic | 7 (2.3%) |
| Monsoon | 90 (29.5%) | Unknown snake | 15 (4.9%) |
| Post monsoon | 79 (25.9%) | **Length of hospitalization** | |
| Winter | 62 (20.3%) | Min-Max | 1-65 |
| Circumstance of Bite | | | |
| Agricultural activity | | - | 83 (27.20%) |
| Playing | | - | 12 (3.90%) |
| At residence | | Activity not specified | 25 (8.20%) |
|  |  | Sleeping | 15 (4.90%) |
|  |  | Household chores | 22 (7.20%) |
| Outside Residence | | - | 17 (5.60%) |
| Research scholar | | - | 1 (0.3%) |
| Collecting firewood | | - | 9 (3%) |
| Construction/ Industrial work | | - | 9 (2.90%) |
| Forestry | | - | 2 (0.70%) |
| Gardening | | - | 8 (2.60%) |
| Walking | | During daytime | 18 (5.90%) |
|  |  | During night | 42 (13.80%) |
|  |  | In the fields | 6 (2.0%) |
| While in pond | | - | 1 (0.3%) |
| Human animal interaction | | Livestock Handling | 1 (0.3%) |
|  |  | Rescuing/Handling Dog | 1 (0.3%) |
|  |  | Rescuing/Handling snake | 6 (2.0%) |
|  |  | Steeped on a snake | 1 (0.30%) |
| Trekking/Fishing | | - | 2 (0.7%) |
| Urinating in open space | | - | 1 (0.30%) |
| Vehicle related activity | | - | 4 (1.30%) |
| Unknown | | - | 19 (6.20%) |

**APPENDIX 5:**

**Definitions of level of healthcare facility**

**Indian Public Health Standards**

1. **Primary healthcare (PHC):** A PHC is the first point of contact between the rural community and a qualified medical officer. It serves a population of about 30,000 in plain areas and 20,000 in hilly or tribal regions. PHCs provide basic curative, preventive, and promotive healthcare services and act as referral units for 5–6 sub-centres. Each PHC typically has 4–6 inpatient beds and is staffed by a medical officer and supporting paramedical staff.

(<https://nhm.gov.in/images/pdf/guidelines/iphs/iphs-revised-guidlines-2012/primary-health-centres.pdf> )

1. **Community health Centre (CHC):** A CHC is a secondary-level healthcare facility that serves about 120,000 people in plain areas and 80,000 in hilly or tribal regions. It functions as a referral center for Primary Health Centres within a block and provides specialist care, including surgery, medicine, obstetrics, and paediatrics. A CHC typically has 30 beds, an operation theatre, X-ray, laboratory, and maternity facilities, and is staffed by four medical specialists and supporting paramedical staff.

(<https://nhm.gov.in/images/pdf/guidelines/iphs/iphs-revised-guidlines-2012/community-health-centres.pdf> )

1. **Taluka /Sub-district Hospital**: A Sub-district/Sub-divisional Hospital is defined as a hospital at the secondary referral level responsible for the Sub-district/Sub-division of a defined geographical area containing a defined population. A Sub-district hospital typically caters to a population of 1,00,000 to 5,00,000, with bed strength ranging from 31 to 100, and provides comprehensive secondary health care services, including specialist and referral services.

(<https://nhm.gov.in/images/pdf/guidelines/iphs/iphs-revised-guidlines-2012/sub-district-hospital.pdf> )

1. **District hospital:** A District Hospital is a secondary referral-level hospital responsible for providing comprehensive healthcare services to a defined population within a district. It serves as a critical component of the district health system, offering curative, preventive, and promotive healthcare services. The hospital caters to both urban and rural populations and functions as a referral center for lower-level public health institutions such as Sub-district Hospitals, Community Health Centres, Primary Health Centres, and Sub-centres. The size of a District Hospital is determined by the population it serves, which can range from 35,000 to 3,000,000

(<https://nhm.gov.in/images/pdf/guidelines/iphs/iphs-revised-guidlines-2012/district-hospital.pdf> )

1. **Private clincis:** Clinics are defined as medical facilities run by a single or group of physicians or health practitioners, smaller than hospitals, and generally providing outpatient services

(<https://clinicalestablishments.mohfw.gov.in/sites/default/files/2021-09/650_1.pdf> )

1. **Private Hospitals:** A private hospital is a non-governmental healthcare institution owned and operated by individuals, partnerships, registered companies, or societies/trusts. These hospitals provide medical services and treatment to patients and are categorized under the "Non-Government/Private" ownership type.

(<https://clinicalestablishments.mohfw.gov.in/sites/default/files/2021-09/650_1.pdf> )

1. **Tertiary Care Centre: A** tertiary care center is defined as a specialized facility that provides advanced management and rehabilitation for complex medical conditions, often utilizing a multidisciplinary approach and emerging therapies, typically located in teaching hospitals or well-equipped institutions.

(<https://www.sciencedirect.com/topics/nursing-and-health-professions/tertiary-care-center> )

**Definitions of Disability**

1. **Renal Failure:** Deterioration of renal function over hours or resulting in the accumulation of toxic wastes and the loss of internal homeostasis, characterized by, Serum creatinine of >4milligrams/dL and acute increase >0.5milligrams/dL OR GRF decrease >75%. Evidence from Priyamvada etal.( <https://doi.org/10.1093/ckj/sfaa168>) shows that snakebite induced AKI often does not fully recover, many survivors demonstrate persistent decline in renal function and progression to chronic kidney disease or even end stage renal disease. The paper’s longitudinal follow-up supports the notion of long term renal impairment as a consequences of severe AKI due to envenomation.

(Tintinalli’s emergency medicine, Judith E. Tintinalli, Eighth edition)

1. **Non-healing ulcer:** Chronic ulcers or non-healing ulcers are defined as spontaneous or traumatic lesions, typically in lower extremities that are unresponsive to initial therapy or that persist despite appropriate care and do not proceed towards healing in a defined time period with an underlying etiology that may be related to systemic disease, local disorders, or specific injuries such as snakebite envenomation.

(<http://pmc.ncbi.nlm.nih.gov/articles/PMC5327512/> ) (<https://doi.org/10.1016/j.toxcx.2021.100074> )

1. **Amputation:** Amputation is defined as the surgical removal of useless or nonviable parts of an extremity that have been severely damaged by disease or trauma, often due to life-threatening infections, irreversible vascular compromise, or extensive tissue damage. Around 81 410 to 137 880 people die each year because of snake bites, and around three times as many amputations and other permanent disabilities are caused by snakebites annually.

([https://www.sciencedirect.com/topics/nursing-and-health professions/amputation](https://www.sciencedirect.com/topics/nursing-and-health%20professions/amputation) )

(<https://www.who.int/news-room/fact-sheets/detail/snakebite-envenoming> )

1. **Exertional pain:** Exertional pain is a general terminology for pain that occurs during or after physical activity exercise.
2. **Dead:** No longer alive.
