# Supplementary material for: Pre-hospital interventions in snakebite: A telephonic survey and follow up investigating snakebite envenoming from a tertiary care centre in Coastal Karnataka
Source: PLoS Negl Trop Dis. 2025 Dec 19;19(12):e0013334. doi: 10.1371/journal.pntd.0013334 (PMC12742758; doi:10.1371/journal.pntd.0013334)
Supplement: Attachment — Submitted filename: Response to Reviewers.docx [file pntd.0013334.s004.docx]

|  | **REVIEWER 1** | |
| --- | --- | --- |
| 1 | Line 186-88: significant association of Tourniquet use indicating better clinical outcomes (Fisher’s Exact Test, p = 0.002) is contradictory with the set up concept of harmful intervention that WHO guidelines highlight. If patients using tourniquet increased full recovery (74.3% vs. 54.9%) with lower mortality (0.5% vs. 4.9%) compared to those who did not, this should be accordingly discussed with additional evidence. Otherwise, this will be questionable. | Justification for this result has been discussed under discussion, but to strengthen the section we have modified the section with some additional references and has been highlighted Line 394-413 |
| 2 | In my understanding, authors retrospectively phone contacted 80 snakebite patients among 273 eligible respondents.  But, in Table 3, authors measured association between pre-hospital interventions adopted by patients and outcomes of treatment at hospital where they received directory of patients' contact details for phone survey. The measure of association including non-patient respondents (eyewitness) would not give detailed information on prehospital care to outcomes to reflect the impact of interventions on outcome. So, author needs to revise the entire data set to re-analyze only real-patients' data (parents in case of deceased cases and children) for impact interventions. | A total of 578 cases were identified retrospectively from the Registry and hospital records during the study period and subsequently contacted for the telephonic survey. Of these, 273 consented to participate in the survey.  The survey was administered to eyewitnesses, reliable informers, or good Samaritans who were directly involved in providing the first response and in transporting the patient to the hospital (as highlighted in Results section, Lines 194-196) . We agree that there may be multiple first responders involved in care of the patient, but we have contacted most reliable informer who is witness/responder and aware of the entire incident.  We would like to clarify that the tern “ first responder” in Table 3 does not refer to the individual responding to the survey call, but rather to the person who provided the initial first aid following the bite. In some cases, this was the patient himself/herself ( e.g., application of tourniquet), regardless of whether the response as medical or non-medical. This has been incorporated in table 3 result discussion and highlighted. Line 202-203. |
| 3 | Similarly, the table 4 is not factual. All eye-witnesses might not accompany the patients. So, the table made based on hearsay information cannot be reliable for scientific interpretation | Information was cross- verified through reliable informers, who were present at the scene or directly involved in the first response and who subsequently accompanied the patient to the hospital, where their details were recorded for communication regarding patient care. In addition, verification was supported by pre-hospital, primary care and tertiary care pre-admission care records from the hospital that patient visited. Patient without such verifiable information or without a reliable informer were excluded from the study. We assure the reviewers that there is no falsification of data, and this is guaranteed by the investigators. Furthermore, relevant hospital records shared by patients were also used to support data accuracy. |
| 4 | Line 142-49: This section needs re-writing. Authors mentioned "no more than 15 minutes" required to get answered for set up questions (Table 1). Below, they highlighted that "Any questions participants had regarding snakebites were thoroughly addressed during the telephonic interview. Their social outreach initiative approach has weakened this section. | Interviews were limited to 15 minutes for collecting incident details and administering the survey questions. As per IEC recommendations, health education was provided in the local language in reasonable time as per their convenience as an ethical outreach component. Any additional participant queries regarding snakebite were addressed thoroughly and at their convenience.  Same has been modified and highlight under Methodology section, line 154-165  Although under this section we have mentioned in limited manner about the questionnaire, the thematic areas of the questionnaire are mentioned in Table 1 in detail and has been submitted as annexure. |
| 5 | Does the analysis presented match the analysis plan- needs statistical Consultation first -Are the results clearly and completely presented?- partly yes. | Analysis presented aligns with the original research plan. Statistical consultation was obtained throughout the study from Dr.Vennila, one of the co-authors and the institutional representative for statistics. All analysis were reviewed and validated under her guidance to ensure methodological and statistical accuracy. |
| 6 | Are the figures (Tables, Images) of sufficient quality for clarity? ## Figures needs improvement with legible texts. | Figures have been replaced with high resolution. |
| 7 | Title: please, remove full stop from the title.  Abstract: Please, replace ";" with "," while listing objectives. The objective "describe the nature of medical and non-medical pre-hospital 26 interventions;" should be further simplified. Please, take care of capitalization throughout the manuscript e.g., A Prospective survey in abstract would be "A prospective survey ...". ..... patients, Tourniquet. See Title of this manuscript.... Similarly, assure a single space between each words, e.g., "3.38.0)and": It would be "3.38.0) and ..". response(70%) .... | Modifications have been made as recommended. |
| 8 | Introduction Authors need to insert citation following up the author guidelines for this journal. Line 72-74: This "Globally, snakebite envenoming occurs in an estimated 1.8 to 2.7 million people annually, resulting in 81,000 to 138,000 deaths and approximately 400,000 cases of permanent disability." needs citation | Citation has been inserted as advised. |
| 9 | Conclusions are not fully supported by the data presented | Conclusion has been modified with key results, line 620-631 |
| 10 | They described limitation in detail but some limitations are so serious that introduced intolerable biases. | We agree that recall bias is a potential limitation in retrospective telephonic survey. However, snakebite is an acute, emotionally intense event, making it more likely that victims and first responders remember the sequence of actions with reasonable accuracy. To further reduce risk of misreporting, responses were cross verified with hospital records, VENOMS Registry and pre-hospital data wherever available. These data provided additional validity to the information collection and helped to offset potential recall related discrepancies.  Limitation section has been modified & highlighted addressing the recall bias concern. Line – 593- 619 |
| 11 | To discuss how these data can be helpful, please, merge the specific recommendations based only on your study findings and discussion in the conclusion section. | This suggestion has has been addressed along with review 9 and highlighted line: 620-631 |
| 12 | Major improvements are needed 305-09: This section needs improvement in support of some educational interventions (published in journals) which increased the prehospital care seeking, such as: Vaiyapuri S, Kadam P, Chandrasekharuni G, et al. 2023. Multifaceted community health education programs as powerful tools to mitigate snakebite-induced deaths, disabilities, and socioeconomic burden. Toxicon: X, 17: 100147. Pandey DP, Thapa CL and Hamal PK 2010. Impact of first aid training in management of snakebite victims in Madi Valley. Journal of Nepal Health Research Council, 8: 5–9. Then, the first two paragraphs should be merged into a single paragraph. | Modifications have been made as advised and has been highlighted.  Suggested studies have been cited and first 2 paragraph has been merged together . |
| 13 | Line 313-39: This section needs rewriting extensively without repeating results. Despite increased reliance on inappropriate first aid practices such as the use of tourniquet and the discussion of association with outcome is not matching. Not only the retrospective nature of study, but also including the past eyewitnesses (with potential memory biases) also misled the findings. So, these findings and association cannot convince the evidence-based readers. | Results from this section have been removed.  We have justified our findings on the association between tourniquet use and outcomes as per the previous comment (1).  Furthermore, this is an IEC approved prospective study and a strict protocol was followed in collecting the data.  Given the intensity of snakebite events, many witnesses tend to have strong recall of circumstances which helps minimize recall bias. This study primarily reflects their perceptions, supported by evidence from the VENOMS Registry and the medical records. |
| 14 | Line 434-36: This section needs to be further highlighted in connection with formal education of first aid in schools and universities in the study regions and in India. In the formal education of first aid, there would be to orient snakebite patients to the government or private facilities where antivenoms are supplied. This would reflect a lack of both formal educaiton and public unawareness. | This section has been addressed as per the suggestions provided and highlighted Line 513-521 |
| 15 | Authors attempted to characterize Pre-hospital Interventions in Snakebite without proper research design and respondents. They need to revise this manuscript after the consultations with experts in the use of dataset to test for hypothesis from this study. There are several errors in English language use throughout the manuscript.  Authors need to find and improve these errors including the structure of English usage. | This methodology was reviewed and approved by the Institutional Research Committee (IRC) and the Institutional Ethics Committee (IEC), and the study has been registered in the Clinical Trials Registry of India (CTRI). The IEC approval is provided in the supplementary material. Statistical analysis was carried out by Dr. Vennila, Associate Professor, Department of Biostatistics, Manipal College of Health Professions, who is also a co-author of this paper.  We have used the Grammarly English correction tool to ensure accuracy of language. |
| 16 | Methods section is inadequate to claim the associations highlighted in the results and discussion section. Authors need statistical consultations before revising methods, results, and discussion section, and resubmitting it. | This is an IRC and IEC approved study, which has already been completed. Therefore, the methods cannot be modified, nor can the results be changes at this stage. The results have been thoroughly verified by the authors, and statistical consultation was obtained both during IRC and IEC phases to ensure methodological rigor and appropriate analysis. Additionally, the results have been reviewed and validated by Dr. Vennila, a professional statistician and co-author of the paper. |
|  | **REVIEWER 2** | |
| 1 | The data on prehospital interventions should not be presented as prospectively collected, as this may be misleading | The VENOMS registry is a prospectively approved registry, however we have sub analysed the data retrospectively, and in order to remove the confusion we have removed the comment to type of study (retrospective or prospective) the VENOMS Registry is and this study is retrospective study that includes data from the VENOMS registry and the medical records. The interview is prospectively conducted. This overall is a mixed method study.  We have made and inserted a flow chart if recruitment process. |
| 2 | Please clearly state the inclusion and exclusion criteria used for participant enrollment | Inclusion and Exclusion criteria’s have been added. |
| 3 | It would strengthen the methodology section to include a flow diagram illustrating the patient recruitment process, starting with the total number of patients registered in the VENOMS registry during the study period, followed by numbers excluded and included, the number of participants who took part in the survey, and the number who did not, along with reasons for exclusion or not participating at each step. | Flow diagram has been added as advised under methodology. |
| 4 | Please specify the timing of participant interviews following hospital discharge. Were the interviews conducted at a standardized time point? The timing could significantly affect reported clinical outcomes and should be clarified | All patients admitted between August 2019, and July 2024 were considered for follow-up. Post-discharge interviews were conducted between October 2024 and March 2025, so the time elapsed since discharge varied across participants. Clinical outcomes, however, were recorded at the time of hospital discharge and not at the time of interview, ensuring that reported outcomes accurately reflect the patients’ condition at discharge rather than at follow-up. This has been incorporated into methodology section. |
| 5 | Please indicate who conducted the participant interviews. This information is important for assessing potential interviewer bias and overall study reliability | This study was conducted as a part of a thesis project by the principal investigator who is the First author here. The use of a structured questionnaire, along with reliance on data sources, strengthens the study’s validity and minimizes potential interview bias. The data collection was carried out with integrity, honesty ad adherence to good ethical conduct.  The same has been justified under the methodology section 2.5 |
| 6 | Providing basic demographic information of the study participants would be valuable for readers to better understand the characteristics of the study population. | Basic demographic information has been added as advised as table 2. |
| 7 | Table 2: It would be more informative to present the first responses in ascending order, starting with the most common. This will help readers quickly identify the most frequently reported responses | Changes have been made as recommended and highlighted in Table 3. |
| 8 | Please clarify which indicators were used to assess hemodynamic status in the study population. This is important for interpreting the clinical relevance of the findings. | Indicators used here are shock and hypoxia. This has been incorporated under the results section. Line 224-227 |
| 9 | Table 3: Consider presenting the total numbers of patients who died, fully recovered, were discharged with disability, or were discharged against medical advice in the relevant cell in the title row. This would improve the clarity and comparability of the outcome data. | Modification done as advised in Table 4 (previously table 4). |
| 10 | Please avoid using non-standard abbreviations such as ED, EMT, ASHA, PHC, ALS/BLS ambulance, etc., or provide a clear legend/footnote defining them. This will ensure a better understanding, especially for international readers. | Changes have been made as advised |
| 11 | Tables 4 and 5: Kindly clarify the meaning of “Nil.” Explicit labeling will reduce ambiguity | ‘Nil’ refers to patients who received definitive treatment and therefore did not visit any further healthcare facility.  To improve the clarity, we have removed the ‘nil’ entries and revised the accompanying text to clarify that among of 273, 87(31.9%) received definitive management at the tertiary care centre (KMC Manipal) and did not proceed to another facility and so on.  Similar modifications has been made for table 5 ( revised to table 7) |
| 12 | Figure 2: This figure does not appear to contribute significantly to the understanding of the results. The authors may wish to reconsider its inclusion and evaluate whether it adds meaningful value to the manuscript | Figure 2 is included to provide a visual representation of geographic hotspots, which is crucial for interpreting whether the lack of ambulance access is an actual logistical issue or primarily a matter of perception. Additionally, the figure helps to highlight areas that require targeted interventions and raises awareness about disparities in pre-hospital care availability, which is a key objective of our study. |
| 13 | Figure 3: This again raises a concern about the timing of post-discharge interviews. What is meant by “current” in this context? Please specify the time elapsed since discharge when participants were assessed, as this can influence their reported knowledge, attitude, and outcomes. | Current refers to the disability reported by participants at the time of interview, reflecting their functional status at that point rather than immediately post-discharge over the course of the study period.  All patients between August 2019 and July 2024, were considered for the follow up. Post discharge interviews were conducted between October 2024 and March 2025, meaning the time elapsed since discharge varied across participants. “current knowledge, attitude and practices refer to the status reported by participants at the time of interview.  This content has been incorporated into methodology section and highlighted. |
| 14 | Please include the guided questionnaire used for assessing current knowledge and attitudes as a supplementary document. This will enhance the transparency and reproducibility of the study. | The guided questionnaire used for assessing current knowledge and attitudes has been provided as a supplementary document.  Additionally, Table 1 in the manuscript summarises the survey questions along with their corresponding thematic categories, facilitating a clear overview of the instrument and its alignment with study objective. |
| 15 | The conclusions are supported by the data presented. However, it would better if the discussion could address the following facts. 1. The relevance of snake repellents in relation to health-seeking behavior is not clearly established in the current manuscript. Please elaborate on how the use or perception of repellents influenced participants’ responses or their decisions to seek health care. If the connection is indirect, consider clarifying or reevaluating its inclusion in the discussion.  2. The survey spans from 2019 to 2024, a period that includes the COVID-19 pandemic (2020–2023). The pandemic significantly disrupted daily lives (occupation, finances, travel and transportation, etc.), healthcare access, healthcare availability and health-seeking behaviors of people in most countries. A recent study from Sri Lanka (“The Impact of the COVID-19 Pandemic and Post-Pandemic Economic Crisis on Snakebite Patterns in Rural Sri Lanka,” Silva et al., 2025) highlights these effects specifically in relation to snakebite victims. It would strengthen your discussion to acknowledge and reflect on how the pandemic may have influenced your study findings, either in a dedicated paragraph in the discussion or as part of the limitations section. | 1. The use or perception of snake repellents was not directly evaluate in our study, and none of the study participants reported repellents as a factor influencing their health seeking decisions. However, we have included a discussion on repellents to highlight the issue of misinformation and misleading claims. Many commercially available product are unverified and marketed publicly without evidence of efficacy, while awareness about their limitations is low. Although these products did not impact our study population, we believe that mentioning them is important to raise public awareness and emphasise the need for regulatory oversight. This has been clarified in the discussion section of the revised manuscript. (Line 454-457)  2. A total of 578 cases were recorded between August 2019 and July 2024., of which 477 cases occurred during the COVID-19 period i.e. 2020-2023 (2020-95; 2021-94; 2022-109; 2023- 179). While our study did not specifically assess the impact of the pandemic on health seeking behaviour, we acknowledge in the revised manuscript that COVID-19 related disruptions may have influenced patterns of healthcare access and management. This has been reflected under limitation section. |
| 16 | It would be better to have an uniformity in table designs as well as in graph designs. | We have revised the manuscript to ensure consistency in the design and formatting of all the tables and figures. |
| 17 | Please indicate the photo courtesy | All clinical images (Fig 6,7,8) included under discussion section are original, captured by VENOMS Registry investigators, after obtaining informed consent from patients or their attendants” – Photo courtesy has been added under methodology/detailed description of data collection section line 165-167 |
|  | **REVIEWER 3** | |
| 1 | The statistical analysis must be better described. Were the data analyzed for normality (which methodology)? When the authors describe results presented as mean, do they mean and SD or SE? Which tests were used to compare quantitative data? | The present study primarily involved descriptive analysis. Continuous variables were summarized as mean ± standard deviation (SD), and categorical variables as frequencies and percentages. Data were checked for normality using the Shapiro–Wilk test. As the objective was descriptive, no inferential statistical tests were applied. Statistical analysis has been revised in the manuscript. Lines: 183-188 |
| 2 | Do the authors have data on the age and sex of the patients | Data on age and gender of the participants has been presented in Table 1. |
| 3 | Do the authors have data on the use of AV? | Data on the use of antivenom was not reliably available from participant interviews. While AV administration records are available in hospital medical records department for all admitted patients, pre-hospital AV data could only be accessed in cases with pre-admission medical records and therefore not included in this study. |
| 4 | Please compare the responders with the non-responders regarding available characteristics. Are the groups different? | Non-responder data with available characteristics has been analysed and provided in supplementary material as annexure 4. |
| 5 | Please provide the definitions used for classifying the order and level of healthcare facility visited, as well as the definitions used for the disabilities described. | The classification of healthcare facilities has been done as per the Indian healthcare system. Definitions of the various levels of healthcare facilities, as well as the classifications of disabilities, along with their references have been provided in the supplementary material (Annexure 5). |
| 6 | Please, clarify if the Institutional Ethical Committee approved the protocol. | Ethics committee clearance has been mentioned under study design line 109-111.  Additionally, approval letter has been submitted Supplementary material as annexure 1. |
| 7 | Please rewrite the discussion, avoiding describing the results again and focusing on comparing the current data with the available evidence and on the implications of the current findings. | The discussion has been modified with available data. |

.
