# Supplementary material for: Pre-hospital interventions in snakebite: A telephonic survey and follow up investigating snakebite envenoming from a tertiary care centre in Coastal Karnataka
Source: PLoS Negl Trop Dis. 2025 Dec 19;19(12):e0013334. doi: 10.1371/journal.pntd.0013334 (PMC12742758; doi:10.1371/journal.pntd.0013334)
Supplement: Attachment — Submitted filename: Response_to_Reviewers_auresp_2.docx [file pntd.0013334.s005.docx]

| **Sl.no.** | **Reviewer comments** | **Response to comments** |
| --- | --- | --- |
| REVIEWER 1 | | |
|  | Are the objectives of the study clearly articulated with a clear testable hypothesis stated? # yes -Is the study design appropriate to address the stated objectives? # No -Is the population clearly described and appropriate for the hypothesis being tested? #. The population described in this manuscript is not appropriate for hypothesis being tested. -Is the sample size sufficient to ensure adequate power to address the hypothesis being tested? # Not sure -Were correct statistical analysis used to support conclusions?  # No -Are there concerns about ethical or regulatory requirements being met? # Not at all.  Does the analysis presented match the analysis plan? # Partly, yes. -Are the results clearly and completely presented? ## yes -Are the figures (Tables, Images) of sufficient quality for clarity? ## yes  Are the conclusions supported by the data presented? ## needs revision still -Are the limitations of analysis clearly described? ## yes -Do the authors discuss how these data can be helpful to advance our understanding of the topic under study? # Yes -Is public health relevance addressed? #Yes  Although authors claimed that analysis presented aligns with the statistical consultation from Dr. Vennila, one of the co-authors and the institutional representative for statistics, I would like to suggest you send it to a statistician for re-review of all analysis to ensure methodological and statistical accuracy.  Now, this article is adequately improved requiring minor revisions. |  |
|  | Authors attempted to retrospectively characterize Pre-hospital Interventions of envenomed victims without proper statistical analysis. They still need to revise this manuscript with/without testing for hypothesis in a non-random dataset obtained from hospital records. For example: their justification for inferential statistics and its interpretation can mislead the results. | We thank the reviewer for highlighting this important concern. Our dataset includes records obtained from the Medical Records Department (MRD) for cases that were not enrolled in the VENOMS registry during the study period. Because these cases represent a non-random subset, we acknowledge that inferential statistics must be interpreted with caution.  We have explicitly stated that non-responders and missing registry enrolments constitute a key limitation of this study  We fully acknowledge that observational datasets may impose limitations on hypothesis testing. To ensure that our analytical approach was appropriate and not misleading, we conducted a post-hoc power analysis using G*Power version 3.1.9.4.  Using the parameters relevant to our test (effect size w = 0.353, α = 0.05, total sample size = 273, df = 3), the computed non-centrality parameter was λ = 34.10, with a critical χ² value of 7.81, resulting in an achieved power of 0.9995. This demonstrates that our dataset had more than sufficient power to detect meaningful associations, and the likelihood of Type II error was extremely low. |
|  | Specific Title: please, remove full stop from the title in the system page of the journal, too. | Changes have been made as advised. |
|  | Introduction Authors still need to insert citation following up the author guidelines for this journal. The mentioned reference number 2 is not primary source to acknowledge for "Globally, snakebite envenoming occurs in an estimated 1.8 to 2.7 million people annually, resulting in 81,000 to 138,000 deaths and approximately 400,000 cases of permanent disability.". So, please, acknowledge the primary sources for the data and text citation throughout this manuscript. Please, check whether you have acknowledged duly to all owners of the article that you have cited in this manuscript. | Primary sources have been citated as advised. |
|  | Methods Method is improved well except the use of inferential statistics to generalize the findings. Please, also mention the duration (MM-YYYY to MM-YYYY) for the telephonic survey in the data collection section. | Changes have been made as advised. |
|  | Results Please, improve the results for tourniquet use without associating with clinical outcomes because of retrospective data, lack of due observational evidence, and non-random dataset. To avoid questionable interpretation, please, be confined with descriptive statistical analysis with improvement in discussion accordingly. | We agree that, given the retrospective nature of the dataset and reliance on telephonic responses, it is not appropriate to draw any causal or outcome-related inferences regarding tourniquet use. In response, we have revised the Results and Discussion sections to ensure that tourniquet data are presented strictly descriptively, without any association with clinical outcomes. |
|  | Next, " Time taken to reach the first healthcare facility:" should be clarified whether those facilities were provided with antivenom and ancillary treatment services.  In most of remote healthcare facilities, only limited amount of antivenom is supplied, which results in administration of limited dose of antivenom and referral to higher center. So, in your comprehensive survey the proportion of such facilities accessed by the enrolled patients would give further clear picture of the scenario of health services and need of improvement. | We agree that the treatment capability of the first healthcare facility—including the availability of antivenom and ancillary services—is an essential contextual factor in interpreting the “time to first healthcare facility” metric. In response, we have added clarifications in the Results section specifying the number of patients who received antivenom at the first healthcare facility (107 of 273).  We have also revised the discussion accordingly. |
|  | Discussion:  Line 392-393: Please, improve these lines with the relevant reference citation as: "Earlier guidelines did recommend the use of tourniquets as a first-aid measure for snakebites (references for those guidelines herein, please); however, with evolving evidence, current protocols (references for those protocols herein, please) no longer advise their use.  Please, improve the discussion about continuity of widely disregarded tourniquet use. This continuity might also be dependent on the erroneous first aid learning in schools and universities of Karnataka like in Nepalese contexts [see the abstract published in Toxicon: Pandey DP, Khanal BP, Sapkota H. 2024. Persistent teaching of harmful interventions for pre-hospital care of snakebites in Nepal. Toxicon 248: 108004 (108038).]. The continued use of tourniquets can be discouraged more effectively through improved formal education in this state of India.  The association of tourniquet use with improved outcomes in a telephonic survey of envenomed patients admitted in Tertiary Care Centre in coastal Karnataka of India could be the resultant of unavoidable limitations in retrospective telephonic data and none of the authors observed them prospectively for the detailed information on tourniquet tightness or time of its application or its effectiveness to occlude blood flow. That is why, this is a serious concern to generalize this finding based on weak study methods. It is because the non-effective (loose) tourniquets do not restrict venom movement. The loose ligature is medically ineffective, but it is less harmful. Therefore, inferential statistical interpretation of these weaker data should not be included herein. Further, systematic review performed in 2016 has highlighted no benefit from tourniquet use in snakebite as a first aid, rather higher risk of local envenoming is reported (please, see the 12th reference). So, it is better to present the retrospective data without performing the inferential analysis. | We thank the reviewers for their valuable comments regarding the discussion on tourniquet use. We agree that the limitations of retrospective telephonic data, including lack of detailed information on tourniquet tightness, duration, and effectiveness, preclude valid inferential analysis. In response, we have revised the manuscript to remove all inferential statistical analysis regarding tourniquet use and have retained only the descriptive results in the discussion section.  As suggested, we have acknowledged the possibility that erroneous first-aid concepts may be propagated through educational or informal learning pathways, and we have referenced the relevant study from Nepal.  We have cited all the relevant references. |
|  | Authors' claim of more than 50% of tourniquet users (Table 5) reaching healthcare within 30 minutes does not suggest improved outcomes. Even patients admitted early in advance discharged with no envenoming and re-admitted after evoluaiton of envenoming after about 26 hours [see the reference: <https://www.researchgate.net/publication/298213568>]. So, early hospitalization alone is not an independent prognosticator in snakebite recovery.  With this weak database for this particular aspect, you can suggest for further study to considered prospective study to achieve statistically acceptable inferences while performing the outcome analysis | Modifications have been made as advised. |
|  | Conclusions "In addition, 22.7% of participants reported ambulance unavailability highlighting the gaps in pre-hospital care access. While some received pre-hospital care, the nature and quality of these interventions was unclear, pointing to the need for standardized protocols". This is part of discussion section. Please, shorten the recommendations and place them under the Conclusion section. | Modifications have been made as advised |
| REVIEWER 2 | | |
|  | 1. Inclusion and Exclusion Criteria: The terminology used to describe the inclusion and exclusion criteria should be made more precise. Based on the information provided, all patients registered in the VENOMS registry were RECRUITED for the study. Among these, only confirmed snakebite patients were included (INCLUSION criteria). Patients who were bitten by other animals or who did not provide consent to participate were excluded (EXCLUSION criteria). It is not necessary to list all the specific animals (non-snakebites) in EXCLUSION criteria list. Please revise the text to ensure consistency and accuracy in the use of these terms | Changes have been made as advised. |
|  | 2. Flow Chart of Patient Recruitment:  The current flow chart depicting patient recruitment is somewhat confusing and should be reorganized for better clarity. Suggested approach: a. Start with the total number of patients registered during the study period in the VENOMS registry. b. Exclude those bitten by animals other than snakes. c. From the remaining group (snakebite patients), exclude those who could not be contacted, followed by those who declined consent. d. The final group—patients who were contactable and gave consent—represents those included in the study. It is not necessary to list all the specific reasons for being uncontactable. Instead, use a broader heading and present the corresponding numbers for clarity. | We would like to thank the reviewer for their valuable suggestion.  We have made modifications to the flowchart as advised. |
|  | In the methodology section, please mention the information obtained by the VENOMS registry. It would prevent the confusion of OUTCOME. (Discharge outcomes and long-term outcomes). Accordingly, change the title and the description of Table 4.  The investigators have responded to most of the comments raised in the previous review. However, several aspects still require attention to enhance the clarity and overall quality of the manuscript which have been mentioned under the comments for methodology. | We have revised the Methodology section to explicitly state that discharge outcome data (including full recovery, disability at discharge, mortality, and DAMA) were extracted from the VENOMS Registry, which records clinical status at the time of discharge from the hospitalization related to the snakebite event. We also clarify that long-term functional status was collected separately through the telephonic questionnaire as “current disability status” at the time of follow-up.  We have modified the title and description of Table 4 to reflect its descriptive nature and specify that it presents Outcomes at Discharge. |
| Reviewer 3 | | |
|  | The text has improved, but it needs a in-depth grammatical revision. There are several mistakes and typos. Please clarify in Tables 4 and 5 where the statistical differences are found. | We have now revised Tables 4 and 5 to clearly indicate the statistically significant differences. Variables with significant associations are marked with the symbol *, denoting p < 0.05. A corresponding footnote has been added to both tables for clarity. No other changes were made to the data.  We have also done the grammatical revision. |
